# The extremely reduced, diverged and reconfigured plastomes of the largest mycoheterotrophic orchid lineage

**DOI:** 10.1186/s12870-022-03836-x

**Published:** 2022-09-20

**Authors:** Yingying Wen, Ying Qin, Bingyi Shao, Jianwu Li, Chongbo Ma, Yan Liu, Boyun Yang, Xiaohua Jin

**Affiliations:** 1 State Key Laboratory of Systematic and Evolutionary Botany, Institute of Botany, Chinese Academy of Sciences, Beijing, 100093 China; 2 School of Life Sciences, Nanchang University, Nanchang, 330031 China; 3 Guangxi Zhuang Autonomous Region and Chinese Academy of Sciences, Guangxi Institute of Botany, Guilin, 541006 Guangxi China; 4 Xishuangbanna Tropical Botanical Garden, Chinese Academy of Sciences, Menglun Township, Mengla County, Yunnan, 666303 China

**Keywords:** Gastrodieae, *Didymoplexis*, Plastomes, GC contents, *ycf1*, Substitution rates

## Abstract

**Background:**

Plastomes of heterotrophic plants have been greatly altered in structure and gene content, owing to the relaxation of selection on photosynthesis-related genes. The orchid tribe Gastrodieae is the largest and probably the oldest mycoheterotrophic clade of the extant family Orchidaceae. To characterize plastome evolution across members of this key important mycoheterotrophic lineage, we sequenced and analyzed the plastomes of eleven Gastrodieae members, including representative species of two genera, as well as members of the sister group Nervilieae.

**Results:**

The plastomes of Gastrodieae members contain 20 protein-coding, four rRNA and five tRNA genes. Evolutionary analysis indicated that all *rrn* genes were transferred laterally and together, forming an *rrn* block in the plastomes of Gastrodieae. The plastome GC content of *Gastrodia* species ranged from 23.10% (*G. flexistyla*) to 25.79% (*G. javanica*)*.* The plastome of *Didymoplexis pallens* contains two copies each of *ycf1* and *ycf2*. The synonymous and nonsynonymous substitution rates were very high in the plastomes of Gastrodieae among mycoheterotrophic species in Orchidaceae and varied between genes.

**Conclusions:**

The plastomes of *Gastrodieae* are greatly reduced and characterized by low GC content, *rrn* block formation, lineage-specific reconfiguration and gene content, which might be positively selected. Overall, the plastomes of Gastrodieae not only serve as an excellent model for illustrating the evolution of plastomes but also provide new insights into plastome evolution in parasitic plants.

**Supplementary Information:**

The online version contains supplementary material available at 10.1186/s12870-022-03836-x.

## Background

Plant cells possess two semiautonomous organelles, plastids and mitochondria, both of which have evolved by endosymbiosis [1, 2]. Plastid genomes (plastomes) of photosynthetic higher plants possess conserved gene contents, with approximately 130 genes encoding approximately 80 proteins, 30 tRNAs, and four rRNAs [3, 4]. The plastomes of photosynthetic higher plants exhibit a conserved structure, characterized by a large single-copy (LSC) region, a small single-copy (SSC) region and two large inverted repeat (IR) regions, which separate the LSC and SSC [3, 5, 6]. In nonphotosynthetic plants (heterotrophic plants), plastomes have been greatly altered in structure and gene content because of the relaxed selection pressure on photosynthesis-related genes, thus providing a unique opportunity for exploring genome evolution under relaxed selection [3, 7–10]. Gene pseudogenization, gene loss and elevated substitution rates are the general trends of plastome degradation in heterotrophic plants [11–13]. The process of plastome degradation, proposed and revised previously, includes the following steps: (1) degradation of photosynthesis and photosynthesis-related genes; (2) degradation of *atp* and housekeeping genes; and (3) nearly complete or complete loss of the plastid genome [10, 12, 14]. Since its publication, this evolutionary model of plastome degradation in parasitic plants has been supported by subsequent studies [10, 12, 14–23].

Mycoheterotrophs are heterotrophic plants that depend on fungi for nutrients and have evolved at least 47 times in land plants [24]. The orchid tribe Gastrodieae is probably the oldest and potentially the largest mycoheterotrophic lineage of the extant Orchidaceae even in land plants, with approximately 100 species [25–34]. Molecular dating indicates that Gastrodieae evolved approximately 35–38 million years ago (Mya) [28, 31], and is possibly one of the oldest groups of mycoheterotrophs in angiosperms [28, 31, 35]. Like most Orchidaceae species, Gastrodieae seeds totally depend on fungal nutrients for germination, but in Gastrodieae and all mycoheterotrophs, this dependence continues throughout their life cycle [10, 20, 36]. One member of Gastrodieae, *Gastrodia elata*, has a long history of use in traditional Chinese medicine [37]. *Gastrodia elata* was successfully cultivated in the 1970s in China and its plant- mycorrhizal interactions, phytochemistry, and medical applications have been intensively studied [38]. The mycoheterotrophic system of Gastrodieae lineage offers a promising model to illustrate the coevolution of mycoheterotrophic plants and their symbiotic microbionts.

Recently, the genomes of two *Gastrodieae* members, *Gastrodia elata* and *G. menghaiensis*, have been sequenced and published [36, 39, 40]. Jiang et al. (2022) reported that the plastomes of *Gastrodia* species have been greatly degraded with the expansion of some nuclear genes encoding plastid proteins, suggesting that plastids play an important role in fully mycoheterotrophic plants [40]. However, little is known about the pattern and mechanism of plastome evolution in this key important lineage. To characterize plastome evolution in this ancient mycoheterotrophic group, we sequenced and analyzed the plastomes of ten Gastrodieae members together with those of its sister group Nervilieae.

## Results

### Molecular systematics of Gastrodieae

**Fig. 1:**
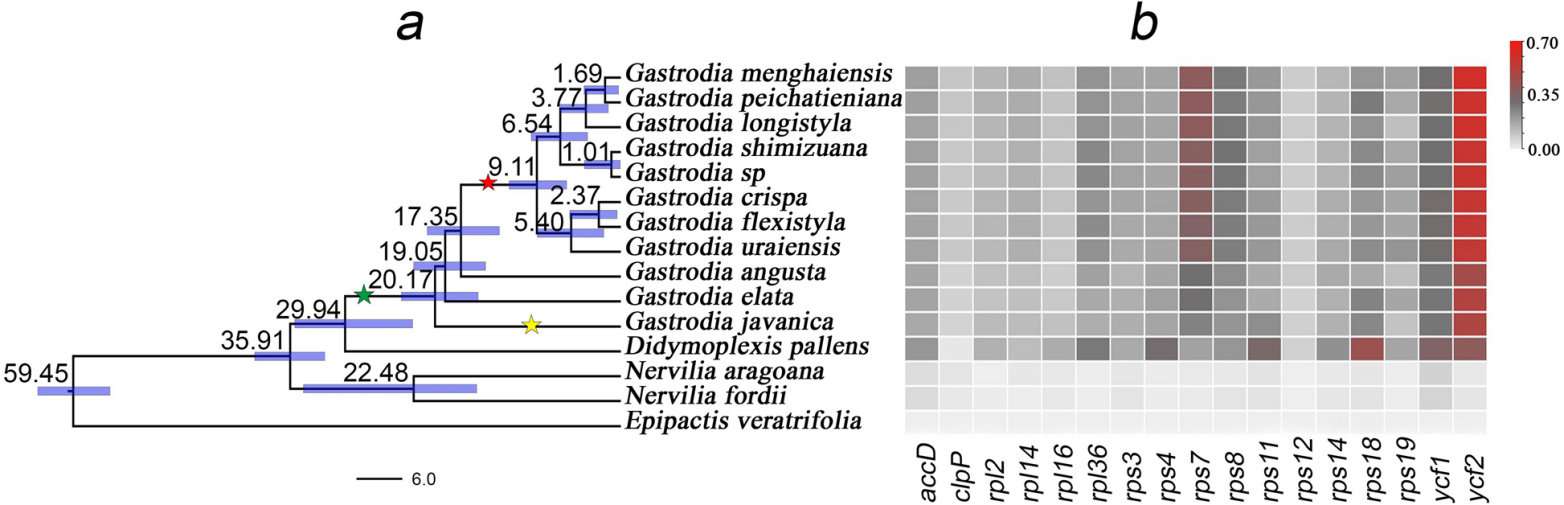
Chronogram and heatmap of nonsynonymous substitution rates (dN) in protein-coding genes. **a** Time-calibrated tree of Gastrodieae. Green star indicates the loss of IR; yellow star indicates the loss of *matK* and *trnW-CCA*; red star indicates the loss of *matK*. **b** Heatmap of dN for each plastid protein-coding gene. Gray and red colors indicate low and high dN values, respectively

Nervilieae is sister tribe to Gastrodieae, which is strongly supported by plastome-based phylogenies (Fig. S1). The genus *Didymoplexis* is sister to the genus *Gastrodia*, with high support, and diverged from *Gastrodia* approximately 29 million years ago (Mya)(Fig. 1a; Supplementary Fig. S1a). Three *Gastrodia* species, including *G. javanica*, *G. elata*, and *G. angusta*, were identified, with high support, as successive sister species to the remaining eight *Gastrodia* species investigated in this study (Fig. 1a). *Gastrodia javanica, G. elata*, and *G. angusta* diverged from the backbone of *Gastrodia* approximately 20, 19, and 17 Mya, respectively, while the remaining eight species formed a tropical clade, which radiated ca. 9 Mya (Fig. 1a). Additionally, *G. javanica*, *G. elata*, and *G. angusta* were characterized by the lack of roots and well-developed tubers and corms, whereas the remaining eight species were characterized by well-developed roots and small black tubers and corms [26, 27, 41].

### Size, gene content, and GC content of Gastrodieae plastomes

**Fig. 2:**
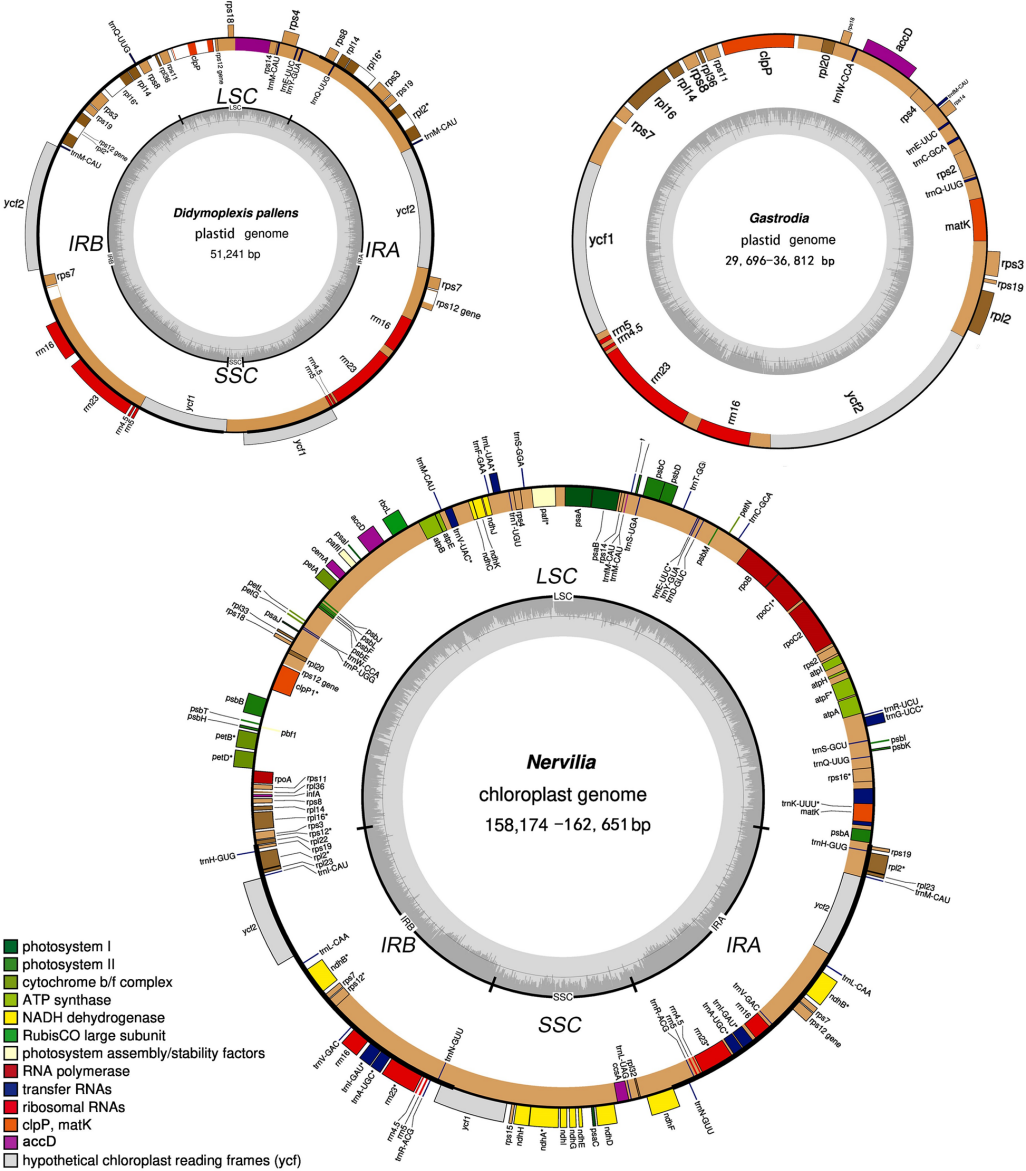
Plastid genomes of *Gastrodieae* and *Nervilia* species

DNA sequencing and assembly revealed that the plastomes of two autotrophic *Nervilia* species (Nervilieae, Orchidaceae) are 15,8174 and 16,2651 bp in size (Fig. 2), while those of species belonging to Gastrodieae varied in length, ranging from 29,696 bp in *Gastrodia peichatieniana* to 51,241 bp in *Didymoplexis pallens* (Table 1, Fig. 2). All *Gastrodia* species showed similar sized plastomes, ranging from 29,696 bp in *G. peichatieniana* to 36,812 bp in *G. angusta.* The plastomes of all Gastrodieae members contained 20 protein-coding genes, four rRNA genes, and five tRNA genes (Table 1). Six housekeeping genes, including *rpl22, rpl23, rpl32, rpl33, rps15,* and *rps16*, appeared to be lost from all Gastrodieae plastomes. The housekeeping gene *matK* was absent from the plastomes of most Gastrodieae members, except *G. angusta* and *G. elata*. Additionally, genes such as *clpP*, *rpl2*, and *rpl16* often contain shorter introns in Gastrodieae plastomes than in Nervilieae plastomes (Supplementary Fig. S2). The *trnW-CCA* gene was lost from the basal branch of *Gastrodia*, *G. javanica*, but was present in remaining members of Gastrodieae, such as *G. elata*, although the *trnW-CCA* gene in Gastrodieae members was approximately 16 bp shorter than its counterpart in *Nervilia* species. The *rrn*4.5 gene in Gastrodieae plastomes contained two 30 bp AT-rich insertions. Secondary structure analyses indicated that this 4.5S rRNA has an altered structure (Supplementary Fig. S3). The plastome of *D. pallens* contained two copies each of *ycf1* and *ycf2*.

**Table 1:** Plastid genomes of Gastrodieae and Nervilieae

Species	Length (bp)				GC content (%)	Voucher	GenBank accession (NCBI)
	Total	LSC	SSC	IR			
*Didymoplexis pallens*	51,241	7,189	3,061	20,495	34.8	Jin X. H. 23332(PE)	ON515488
*Gastrodia angusta*	36,812				25.4	Jin X. H. 17853 (PE)	ON515479
*Gastrodia crispa*	30,582				25.7	Jin X. H. & Arief H. PE-BO 4014 (PE)	ON515481
*Gastrodia elata*	35,304				25.3	Jin X. H. 17638(PE)	MF163256
*Gastrodia flexistyla*	30,797				25.4	Huang Y.S. QY20190302001(IBK)	ON515480
*Gastrodia javanica*	31,896				24.8	PE-BO 4091 (PE)	ON515482
*Gastrodia longistyla*	30,464				26.8	Jin X. H. 25023 (PE)	ON515483
*Gastrodia menghaiensis*	30,158				26.8	Jin X. H. 18195 (PE)	ON515489
*Gastrodia peichatieniana*	29,696				25.9	Jin X. H. 31639 (PE)	ON515484
*Gastrodia shimizuana*	30,019				25.5	Huang Y.S. QY20190226001 (IBK)	ON515485
*Gastrodia* sp. (near *Gastrodia crispa*)	29,944				25.8	Jin X. H. 38054 (PE)	ON515486
*Gastrodia uraiensis*	30,746				24.9	QY1007 (IBK)	ON515487
*Nervilia aragoana*	162,651	91,150	18,603	26,449	36.7	Jin X. H. 23240 (PE)	ON515490
*Nervilia fordii*	158,174	86,875	18,079	26,610	36.8	Jin X. H. 23386 (PE)	ON515491

The plastomes of autotrophic *Nervilia* species showed a typical quadripartite structure (Fig. 2). On the other hand, the plastomes of *Gastrodia* species showed a specialized structure with only one IR region (Fig. 2). All *rrn* genes joined together to form the *rrn* block in plastomes; *rpl* and *rps* genes formed the *rpl-rpls* block, while the four *trn* genes and two to three coding sequences (CDSs) were embedded in the *rpl-rps* block (Fig. 2). The *rrn* and *rpl-rps* regions were separated by *ycf1* and *ycf2*. The highly reduced plastome of *D. pallens* showed a quadripartite structure, with transversion and expansion of IR regions (Fig. 2, Supplementary Fig. S1b). The IR region was extended to a length of 21 kb and contained *rps3*, *rpl16*, *rpl14*, and *rps8* genes. By contrast, the SSC region was reduced to an approximately 3 kb sequence containing no gene. The transversion occurred between *rps4* and *rps14*. Another transversion was observed at a location that coincided with the loss of *trnW-CCA* in *G. javanica* plastome (Fig. 1b).

**Fig. 3:**
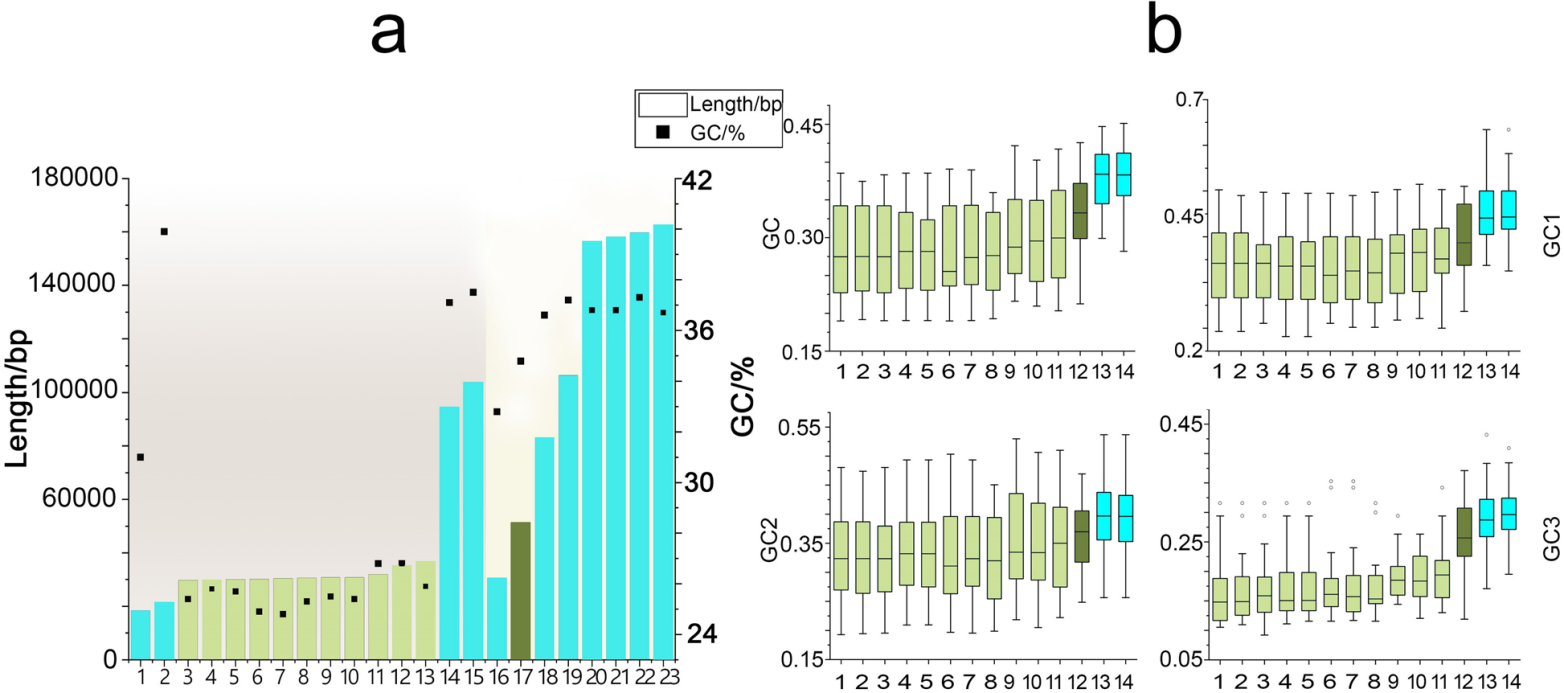
GC content of the plastomes of Gastrodieae and other species. **a**, Relationships between the length and GC content of plastomes of species. Gastrodieae species, green colour. *Gastrodia* species, light green. *Didymoplexis pallens,* dark green. Other species, blue colour. 1: *Epipogium roseum*; 2: *Sciaphila densiflora*; 3: *Gastrodia peichatieniana*; 4: *Gastrodia* sp.; 5: *Gastrodia shimizuana*; 6: *Gastrodia menghaiensis*; 7: *Gastrodia longistyla*; 8: *Gastrodia crispa*; 9: *Gastrodia uraiensis*; 10: *Gastrodia flexistyla*; 11: *Gastrodia javanica*; 12: *Gastrodia elata*; 13: *Gastrodia angusta*; 14: *Aphyllorchis montana*; 15: *Petrosavia stellaris*; 16: *Epipogium aphyllum*; 17: *Didymoplexis pallens*; 18: *Neottia acuminata*; 19: *Neottia camtschatea*; 20: *Cymbidium bicolor*; 21: *Nervilia aragoana*; 22: *Epipactis veratrifolia*; 23: *Nervilia fordii*. **b** Overall GC content and GC content of the first, second, and third positions of codons (GC1, GC2, and GC3, respectively) in the plastomes of various species. 1: *Gastrodia menghaiensis*; 2: *Gastrodia peichatieniana*; 3: *Gastrodia longistyla*; 4: *Gastrodia shimizuana*; 5: *Gastrodia* sp.; 6: *Gastrodia crispa*; 7: *Gastrodia flexistyla*; 8: *Gastrodia uraiensis*; 9: *Gastrodia angusta*; 10: *Gastrodia elata*; 11: *Gastrodia javanica*; 12: *Didymoplexis pallens*; 13: *Nervilia aragoana*; 14: *Nervilia fordii*

The GC contents of plastomes varied greatly in *Gastrodieae* and *Nervilieae*. With total GC contents ranging from 23.10% in *G. flexistyla* to 25.79% in *G. javanica,* the average GC content of eleven *Gastrodia* species was approximately 10% lower than that of autotrophic species, such as *Cremastra* (Orchidaceae) [22], *Holcoglossum* (Orchidaceae) [42], *N. aragoana*, *N. fordii* and *Tipularia* (Orchidaceae) [22] (Table 1, Fig. 3a, Supplementary Table S1). However, the GC content of the *D. pallens* plastome was 34.8%. In the autotrophic Nervilieae species, the GC content was approximately 30% in most CDSs, and up to 44% in genes such as *psbA*, *psbB*, and *psbC*. In *Gastrodia* species, the GC content was approximately 30% in seven genes, including *clpP, rpl2,* and *rpl14*; less than 30% in the remaining 12 CDSs; and less than 20% in *ycf1* and *ycf2* (Supplementary Tables S2). The GC content of *matK* was approximately 21% in *G. elata* and *G. angusta*, and 30% and 32% in the two Nervilieae species (Supplementary Tables S2 and S3). The GC content of the third position of codons (GC3) varied greatly among the three genera investigated in this study: 15–17% in *Gastrodia*; 25% in *D. pallens*; and 27% in autotrophic *Nervilia* species (Table 1, Fig. 3b). Notably, GC3 was less than 10% in *rps18* in *G. longistyla*. Codon usage analysis showed that AAA (encoding Lys) was the most used codon in Gastrodieae, followed by AUA (encoding Ile) and AAU (encoding Asn) (Supplementary Table S3). However, in the autotrophic Nervilieae species, AAU and GAA (encoding Glu) were identified as the two most commonly used codons (Supplementary Table S3).

### Molecular evolution of Gastrodieae plastomes

**Fig. 4:**
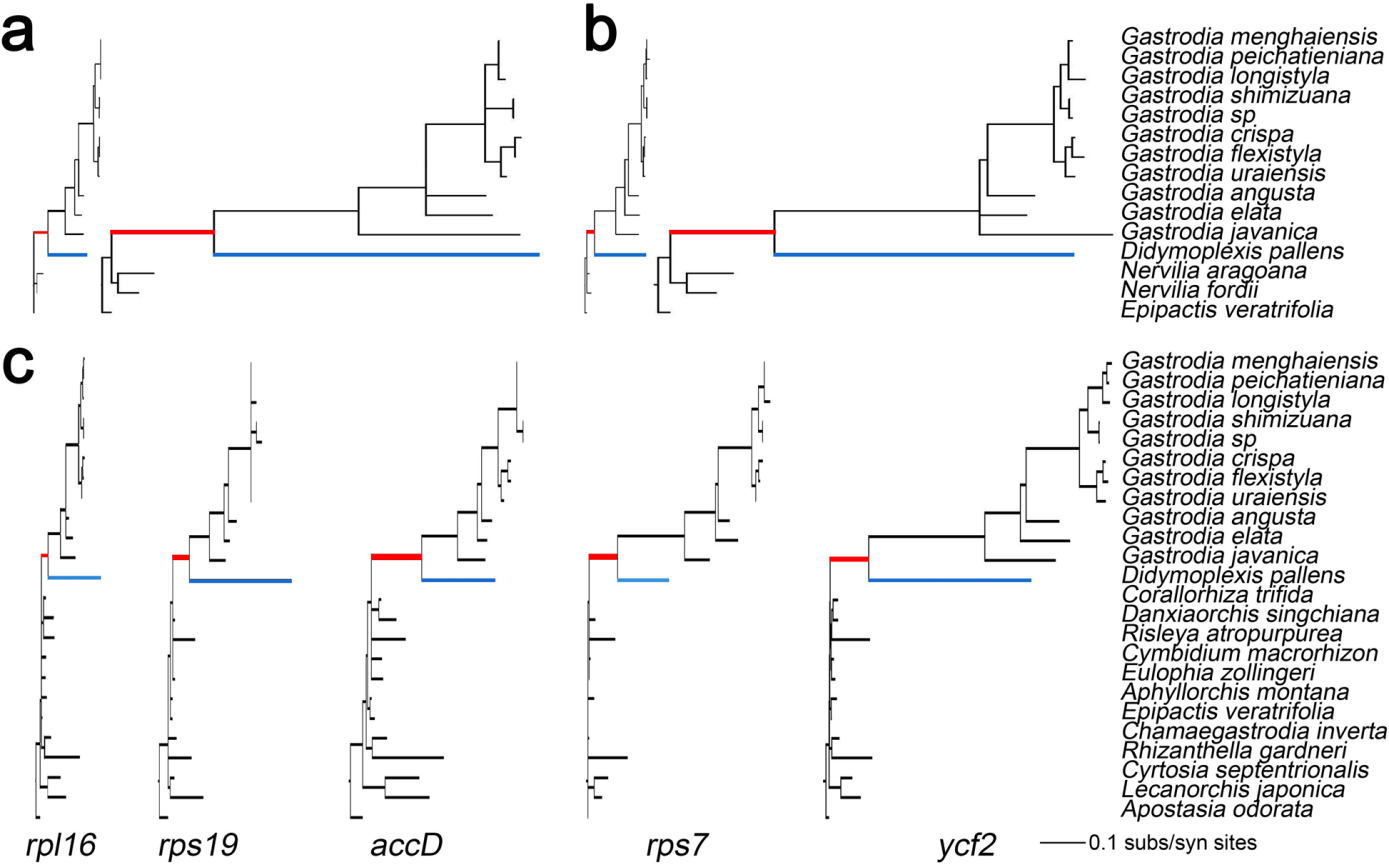
Variation in the values of relative and absolute synonymous substitution rates (dS) in plastid protein-coding genes in Gastrodieae. Red and blue branches show the evolution rates of Gastrodieae species and *Didymoplexis pallens*, respectively. **a** phylograms of dN and dS of *rpl14*, respectively. **b** phyograms of dN and dS of *rps3*, respectively. The value of dS was approximately 10-fold higher than that of dN. **c** phylograms of dN divergence of five individual genes. All trees are drawn to the same scale

Among the mycoheterotrophic species in Orchidaceae, the Gastrodieae species showed especially high synonymous substitution rate (dS) and nonsynonymous substitution rate (dN) in plastomes (Supplementary Figs. 1, 4, 5; Supplementary Table S4). The values of dN and dS in Gastrodieae plastomes were 8–10 times higher than those in the closely-related autotrophic species *Nervilia aragoana* and *N. fordii* (Fig. 4, Supplementary Table S4). The values of dN and/or dS varied across species and genes. The value of dS in four genes, including *rpl14*, *rps11*, *rps18*, and *ycf1*, was very high. However, dS in *rpl36* was very low in Gastrodieae and very high in *D. pallens*. Additionally, the value of dS in four genes (*accD*, *rpl36*, *rps11*, and *rps18*) was approximately 2–4 times higher in *D. pallens* than in *Gastrodia* (Supplementary Fig. S4). Two genes, *ycf2* and *rps7*, showed rather high dN in Gastrodieae, and *ycf2* was under positive selection (Fig. 1b, Supplementary Fig. S4, Supplementary Table S5). The value of dN in *rps7* was considerably high in *Gastrodia* but very low in *D. pallens*. Two genes*, clpP* and *rps12*, showed the lowest substitution rates in Gastrodieae (Fig. 1b).

Based on branch length, the dN and/or dS values changed over time in the various clades. Values of dN in three genes (*rpl36, rps7,* and *rps11*) were low in *G. angusta* but high in its sister group, the tropical *Gastrodia* clade (Figs. 1 and 4). The dS values in *rpl36*, *rps12*, and *rps19* were low in *G. javanica* but high in the remaining *Gastrodia* species (Supplementary Fig. S4). Values of dN and dS in the majority of remaining housekeeping genes, including *accD, clpP, rpl12, rpl14, rps11, rpl16, rpl36, rps3,* and *rps4*, were significantly higher in *D. pallens* than in other Gastrodieae species (Figs. 1 and 4, Supplementary Figs. S4 and S5). RELAX analyses indicated that two genes, *accD* and *ycf1*, were under significant intensification selection (Supplementary Table S6); however, intensification selection pressure on the remaining genes was not significant (Supplementary Table S6). Three genes, *ycf1*, *ycf2*, and *rps3*, were under positive selection in Gastrodieae (Supplementary Table S7).

## Discussion

Characteristics such as small size, very low GC content, and loss of many housekeeping genes indicate that the plastomes of Gastrodieae are highly reduced and have reached the stage of a minimal plastome. However, recent analyses of nuclear genes encoding plastid proteins (NEPs) in *G. elata* and *G. menghaiensis* indicate that many genes involved in the biosynthesis of essential compounds, such as aromatic amino acids (such as L-tryptophan) and fatty acids, have undergone expansion [40]. These findings suggest that plastids play an important role in fully mycoheterotrophic species, despite the loss of photosynthesis [40]. The loss of housekeeping genes in plastomes and expansion of some NEPs define a paradox, which indicates that plastomes of Gastrodieae may still be in the process of reaching stability. The plastomes of Gastrodieae are an excellent model for illustrating the evolution of plastomes, and provide new insights into plastome evolution in parasitic plants.

The plastomes of Gastrodieae are collinear with those of autotrophic Nervilieae species and other autotrophic orchids (Supplementary Fig. S1b); however, there have been several reconfigurations, including the formation of the *rrn* block and loss or expansion of the IR regions. The *rrn* block evolved independently in *Epipogium* (Orchidaceae) [43], Gastrodieae (Orchidaceae), *Rhizanthella* (Orchidaceae) [44], and *Sciaphila* (Triuridaceae) [45]. The convergent evolution of the *rrn* block in these four distant plant lineages indicates that the *rrn* block evolved independently and was positively selected. In this study, analysis of the transcriptome data of *G. elata* downloaded from NCBI (SRR18147619) indicated that at least three blocks, including *rrn*, *clpP-rps11-rpl36-rps8*, and *rpl14-rpl16*, were transcribed together as a single transcript (Supplementary Fig. S6). Jiang et al. (2022) indicated that NEPs of plastid ribosome large subunit underwent expansion [40]. This reconfiguration of plastome structure, transcription pattern of the *rrn* block, and expansion of NEPs of plastid ribosome large subunit may accelerate ribosome assembly, protein translation and biosynthesis of important compounds. This may be related to the special lifestyle of Gastrodieae. Plants of Gastrodieae species grow underground for approximately 3–4 years. However, following inflorescence emergence from the ground, plants grow rapidly to a height of up to 150 cm and disperse seeds within 1 month, thus requiring support from plastid protein function. The reconfiguration of IR regions is common among parasitic species but shows lineage-specific trends [43, 45]. Two extreme trends of IR reconfiguration were observed in this study: (1) complete loss of one IR region in all *Gastrodia* species; and (2) expansion of IRs, spanning 80% or more of the plastome, in the *D. pallens* clade.

Increase in AT content of plastomes is considered as indicator of plastome degradation in heterotrophic plants compared with autotrophic species, and the level of AT-richness somewhat correlates with the degree of plastome reduction [11, 14, 46]. Extremely high AT content has been recorded in two heterotrophic lineages, *Thismia* (Thismiaceae) [46] and Balanophoraceae [47]. Although many species possess highly reduced plastomes, such as *Epipogium aphyllum* (18,339 bp) (Orchidaceae) and *Sciaphila densiflora* (21,485 bp) (Triuridaceae), their GC content is no less than 30% [43, 45]. Both *Gastrodia* and *Didymoplexis* are fully mycoheterotrophic genera in the Gastrodieae tribe; however, *Gastrodia* species have a very low GC content even compared to mycoheterotrophic orchids (Supplementary Table S8), whereas *D. pallens* shows a rather high GC content (34.8%). The high GC content of *D. pallens* might have been contributed by its genome structure and corresponding adaptive changes. Due to the expansion of IRs, the plastome of *D. pallens* contains 44 genes, however, there are 28 to 29 genes in plastomes of *Gastrodia* (Supplementary Table S8). GC content in nontranscribed spacers tends to be considerably lower than elsewhere in the plastome [11, 12]. IR also greatly reduces the substitution rate of genes within IR region [48].

Wicke et al. (2016) suggested that low GC content correlates with increases in the number of structural rearrangements [13]. The 30 bp AT-rich insertions in *rrn4.5* not only make it very difficult to predict the *rrn4.5* [26, 36] but also present a strategy for structural rearrangements that increase the AT content of plastomes. However, the insertion of long AT-rich sequences may lead to the pseudogenization of *rrn4.5*. This bias toward AT-richness is lineage specific, and seems to have evolved after the divergence between *Gastrodia* and *Didymoplexis*. The AT-rich insertion in *rrn4.5* has also been reported in *Balanophora* (Balanophoraceae) [47]. While most members of Gastrodieae lost *matK* during evolution, the low GC content of *G. elata* and *G. angusta* plastomes, which contain *matK*, indicates that *matK* might soon be lost from these two plastomes. The mechanism underlying the adaptation to this bias toward AT-richness remains to be illustrated.

Substitution rates are often elevated in the plastomes of parasitic plants [13, 49, 50]. Wicke et al. (2016) suggested that the elevation of substitution rates in parasitic plants was caused first by relaxed selection and then by rate deceleration due to intensified selection [13]. Our results indicated that *Gastrodia* is one of the Orchidaceae genera with the highest substitution rate, which is approximately 10-fold higher than that of two autotrophic species of *Nervilia* [12]. Some housekeeping genes, such as *accD* and *ycf1*, were under significant intensified selection in *Gastrodia*. However, most genes showed very high substitute rates. Jiang et al. (2022) indicated that some NEPs, including genes encoding plastid ribosomal subunits and *accD*, underwent expansion in *Gastrodia* genomes [40], which suggests that coevolution of the nuclear genome and plastome might have large effects on the molecular evolution of plastid genes. Although the plastomes of *Epipogium* (Orchidaceae), *Gastrodia* (Orchidaceae), and *Thismia* (Thismiaceae) are minimal and at the final stages of degradation, it seems that these plastomes still have very high substitute rates [43, 46]. Recent molecular dating indicated that the fully mycoheterotrophic lineage *Thismia* is of a much more recent origin [43]. All Gastrodieae members are mycoheterotrophic and diverged from their autotrophic relatives (*Nervilia* species) approximately 35 Mya. This suggests that the relaxed selection pressure on plastome genes may last longer than expected.

**Fig. 5:**
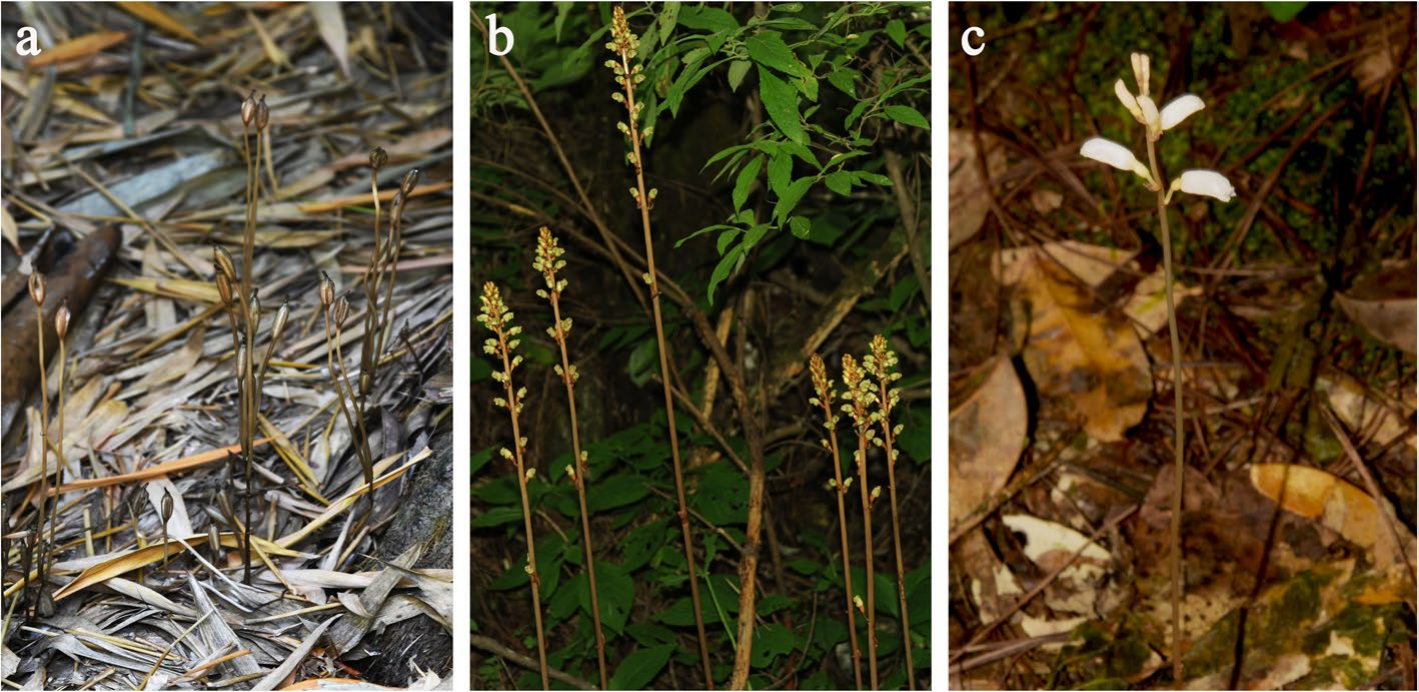
Natural habitat of Gastrodieae species. **a** *Didymoplexis pallens* in dry and open forest*.* b and c, *Gastrodia elata* (**b**) and *Gastrodia menghaiensis* (**c**) in shady and humid forest. Photographed by X.H. Jin

Gene pseudogenization and gene loss are common phenomena in the plastomes of parasitic plants [43, 51]. The *ycf1* gene was often absent from the highly reduced plastomes of parasitic plants growing in humid and shaded environments, such as *Epipogium* [43], *Sciaphila* [45, 52], and *Thismia* [46], as well as from the plastomes of aquatic plants including all members of the Podostemaceae family [53]. To our knowledge, *D. pallens* is the only fully mycoheterotrophic species with two copies of *ycf1* in plastomes*.* In contrast to most mycoheterotrophic plants that grow in humid and shady environments (Supplementary Table S9), our botanical survery indicated that *D. pallens* grows in very dry and open environments (Fig. 5). Although previous results suggest that environment has little effect on plastome evolution, the presence of a duplicate copy of the *ycf1* gene in *D. pallens* and absence of *ycf1* in Podostemaceae suggest that environmental factors may affect the loss, retention, and duplication of genes in the plastomes of parasitic plants in extremely environmental conditions.

## Conclusions

The plastomes of Gastrodieae are greatly reduced and characterized by low GC content, *rrn* block formation, and lineage-specific reconfiguration and gene content. Synonymous and nonsynonymous substitution rates are much higher among the plastomes of Gastrodieae than among those of mycoheterotrophic species in Orchidaceae. Overall, plastomes of Gastrodieae not only serve as an excellent model for illustrating the evolution of plastomes but also provide new insights into plastome evolution in parasitic plants.

## Methods

### DNA extraction and sequencing

A total of 13 species belonging to the Gastrodieae tribe (*Didymoplexis pallens* and ten *Gastrodia* species) and its sister tribe, Nervilieae tribe (two *Nervilia* species), were sampled (Supplementary Table S1) based on previous results [28, 31]. Genomic DNA was extracted from these species using silica-dried materials with the modified cetyltrimethylammonium bromide (CTAB) method [54]. DNA was sheared to 400–600 bp fragments using Covaris M220. DNA libraries were prepared using the NEBNext Ultra DNA Library Prep Kit (New England Biolabs, USA), according to the manufacturer’s instructions, and then outsourced to Majorbio Company (Beijing, China) for 100 or 150 bp paired-end sequencing on the Illumina HiSeq 2500 platform. Approximately 5 Gb of raw data were generated for heterotrophic species, and 3 Gb for autotrophic species. *Epipactis veratrifolia* was used as an autotrophic outgroup for comparative analyses. One plastome downloaded from NCBI (https://www.ncbi.nlm.nih.gov/) were included in the analyses (Supplementary Table S1). In addition, 19 plastomes representing 19 mycoheterotrophic orchid genera were downloaded from NCBI (https://www.ncbi.nlm.nih.gov/) for comparison (Supplementary Table S8).

### Plastome assembly and annotation

Raw reads were trimmed and filtered using NGSQCTOOLKIT v. 2.3.3 [55]. Plastomes were assembled using GetOrganelle v. 1 [56] and NOVOPlasty [57], with default parameters, and the plastome of *Calanthe triplicata* (NC_024544.1) was used as a reference. Contigs were combined and extended using Geneious Prime (Biomatters, Inc., Auckland, New Zealand; http://www.geneious.com) to obtain the plastome draft. Assembly errors were corrected in Geneious Prime by mapping reads to the plastome draft. The boundaries of IR regions in each plastome were confirmed by BLAST. Completed plastomes were annotated with PGA [58] using the annotated plastome of *C. triplicata* (NC_024544.1) as a reference. Then, the annotations were manually checked, and gene or exon boundaries were adjusted using Geneious Prime.

### Phylogenetic analysis and molecular dating

All protein-coding sequences in plastomes were used to reconstruct the phylogenetic relationships (Supplementary Table S1). A single gene matrix was aligned using MAFFT under the automatic model selection option [59, 60] with manual adjustments in BioEdit. Then, each matrix was combined into a single plastome supermatrix using SEQUENCEMATRIX v1.7.8 [61]. The concatenated sequences were analyzed using RAxML [62] in CIPRES [63], with the best-fit model GTRGAMMA. Branch support was evaluated by 1,000 bootstrap replicates. Molecular dating was conducted with the combined supermatrix using BEAST v. 2.1.3 [64–66]. Priors were placed on the stem node of Nervilieae and Gastrodieae (offset: 34.93 Mya; sigma: 1.0) and *Epipactis* and Gastrodieae + Nervilieae (offset: 60.3 Mya; sigma: 1.0), based on previous results [28, 67–69]. Two runs of MCMC searches were performed for 200 million generations with sampling every 10,000 generations, and typically four non-independent chains were used for each run. A Yule process was chosen for the tree prior. Log files were monitored using Tracer v1.6 [70]. The first 10% of trees saved from the first run and the first 8% of trees saved from the second run were discarded, and the remaining trees were combined in Logcombiner v. 2.3.0. Convergence was determined based on the effective sample sizes (ESSs) of all parameters, assessed as more than 100. A maximum clade credibility (MCC) chronogram was generated in TreeAnnotator v. 1.8.0 [64] with median heights for node ages.

### Molecular evolutionary analyses

The CDSs of 17 protein-coding genes common to both Gastrodieae and Nervilieae tribes (Table 1 and Supplementary Table S5, Supplementary Material online) were aligned at the codon level using MUSCLE, with the option “-codon”, in MEGA v. 7.0.2 [71]. Stop codons were removed from the CDSs prior to alignment. The phylogenetic analysis-generated phylogram based on all CDSs was used for evolutionary analysis. The plastome of *Apostasia odorata* (NC_030722.1) was used as a reference. The values of dS and dN in the 17 concatenated protein-coding genes were calculated using CODEML in the PAML v.4.8 software package [72, 73]. The relative values of dS and dN in each CDS were calculated using the pairwise model in the PAML software package [73]. The plastome of *Epipactis veratrifolia* (NC 030708.1) was used as a reference. Selective regimes among branches were analyzed in PAML v.4.8 using the CODEML module [72, 73]. Differences in substitution rates were specifically tested between Gastrodieae and the autotrophic outgroup, and between Gastrodieae + Nervilieae and the autotrophic outgroup. To determine the relative dN/dS ratio in Gastrodieae among orchids, the substitution rates in CDSs were analyzed in 24 representative mycoheterotrophic species across Orchidaceae (Supplementary Table S9). A total of 17 CDSs common to these mycoheterotrophic species were analyzed as described above. To determine whether the relaxed selection on plastome genes varied with the species lifestyle, the variation in selection pressure on these 17 genes was analyzed using RELAX [74]. Gastrodieae and autotrophic *Nervilia* species were treated as different test branches.

Codon usage, amino acid frequencies, and GC3 value in the 12 Gastrodieae and Nervilieae plastomes were calculated using CondonW v1.4.2 (http://codonw.sourceforge.net/), based on the subset of 17 common protein-coding genes. Genes were categorized into groups according to gene function or subunits that form a functional protein complex, as described previously [75]. Statistical analyses were performed using the R software package (http://www.r-project.org), and correction for multiple comparisons was conducted using the Benjamini and Hochberg method (1995), which controls for the false discovery rate. RNAs of various species were compared using Geneious10.2.3, and the secondary structure of RNA was determined using the online software (http://rna.tbi.univie.ac.at//cgi-bin/RNAWebSuite/RNAfold.cgi). Transcriptome data of *G. elata* (SRR18147619) was downloaded from NCBI (https://www.ncbi.nlm.nih.gov/) and analyzed as described previously [36].

## Supplementary Information

**Additional file 1.**

## Data Availability

All newly sequenced and annotated plastid genomes generated in this study have been submitted to NCBI (https://www.ncbi.nlm.nih.gov/) with accession number from ON515479 to ON515489 (Table 1). All plastid genome assembly and annotation data are publicly available (https://www.ncbi.nlm.nih.gov/). The online resources of genomic data were downloaded from NCBI (https://www.ncbi.nlm.nih.gov/), including transcriptome data of *Gastrodia elata* (SRR18147619) and plastid genomes with GenBank accession numbers listed in Supplementary Table S8 and S9.

## Abbreviations

IR: Inverted repeat
LSC: Long single-copy
SSC: Short single-copy
ML: Maximum-likelihood
CDS: Coding sequences
Ma: Million years ago
