# Supplementary material for: The extremely reduced, diverged and reconfigured plastomes of the largest mycoheterotrophic orchid lineage
Source: BMC Plant Biol. 2022 Sep 20;22:448. doi: 10.1186/s12870-022-03836-x (PMC9487142; doi:10.1186/s12870-022-03836-x)
Supplement: Supplementary file 1 — Additional file 1. [file 12870_2022_3836_MOESM1_ESM.zip › 3836_ESM files/Figures S1-6.docx]

**The extremely reduced, diverged and reconfigured plastomes of the largest mycoheterotrophic orchid lineage**

Yingying Wen^1,2,#^, Ying Qin^3, #^, Bingyi Shao^1^, Jianwu Li^4^, Chongbo Ma^1^, Yan Liu^3,*^, Boyun Yang^2,*^, Xiaohua Jin^1,*^

^1^State Key Laboratory of Systematic and Evolutionary Botany, Institute of Botany, Chinese Academy of Sciences, 100093 Beijing, China.

^2^School of Life Sciences, Nanchang University, Nanchang 330031, China.

^3^Guangxi Institute of Botany, Chinese Academy of Sciences, Yanshan, Guilin, Guangxi, China.

^4^Xishuangbanna Tropical Botanical Garden, Chinese Academy of Sciences, Menglun Township, Mengla County, Yunnan 666303, China.


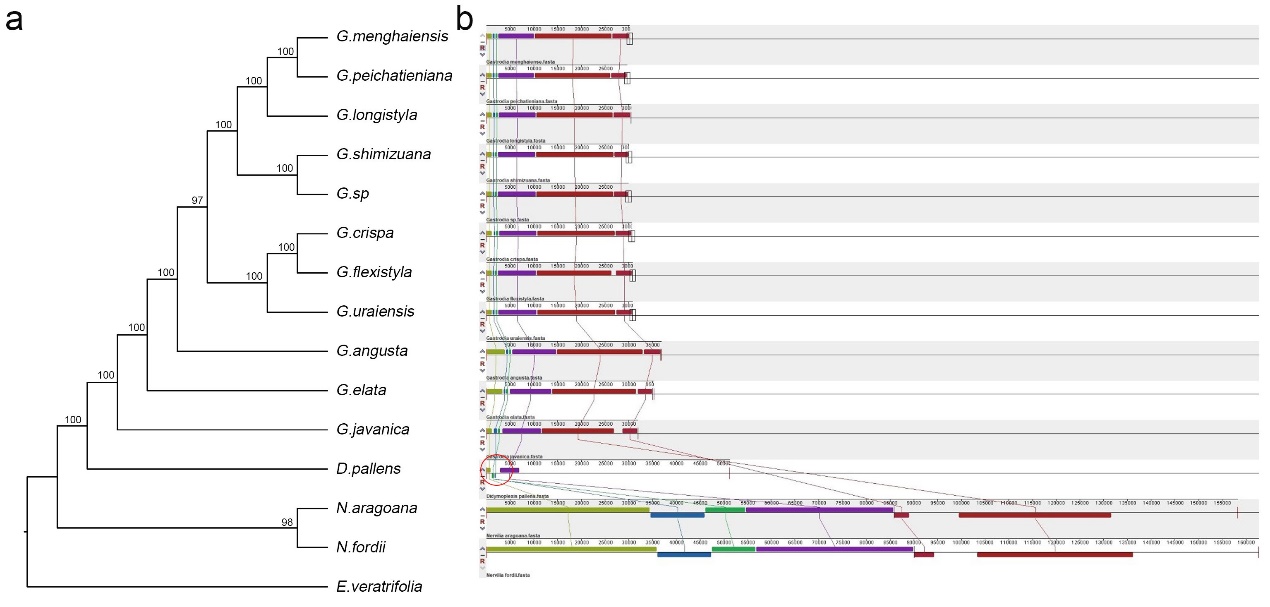


**Supplementary Fig. S1** a, Phylogram based on plastid genomes. b, colinear analysis of plastomes of the Gastrodieae and Nervilieae. Color bands are locally-collinear blocks, representing homologous gene clusters. Red circle shows that the *Didymoplexis pallens* has been inverted.


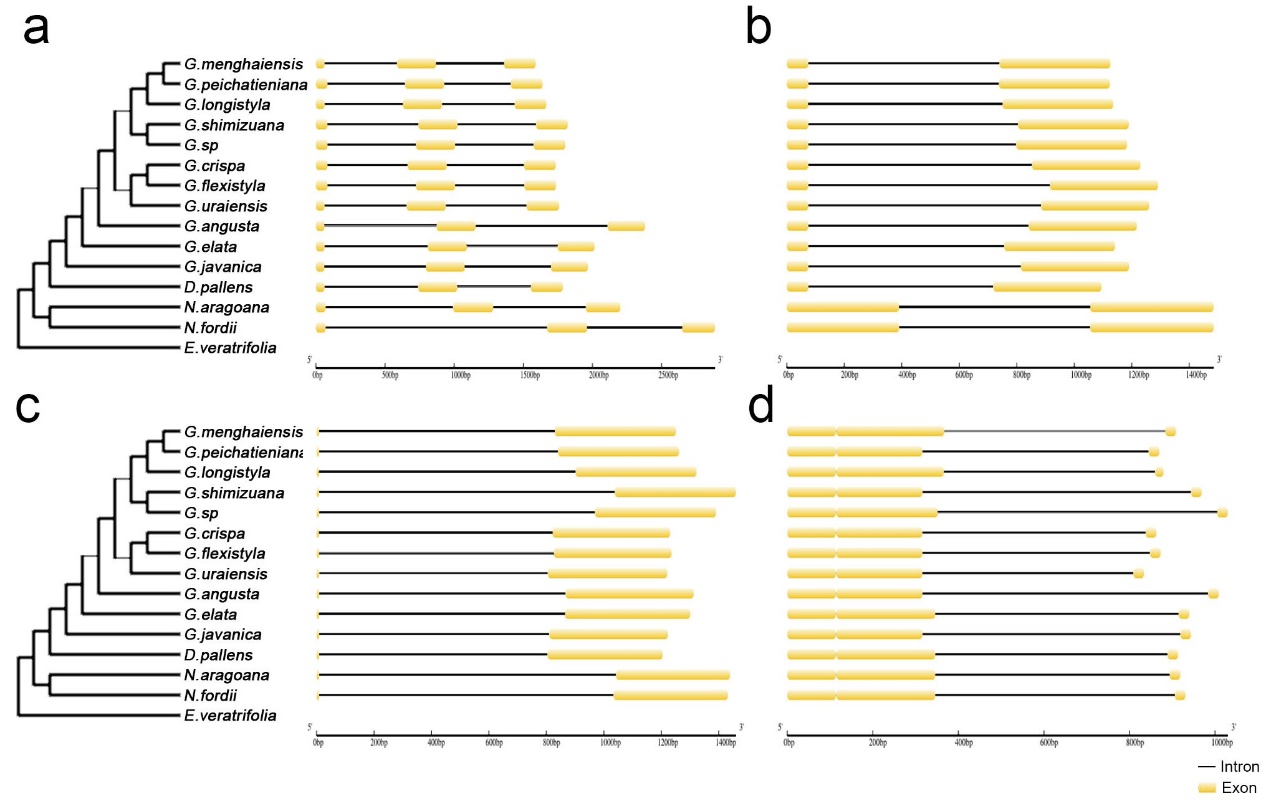


**Supplementary Fig. S2** Differences between introns and exons of four genes shared by Gastrodieae and Nervilieae plastomes. a, *clpp*. b, *rpl2*. c, *rpl16*. d, *rps12*.


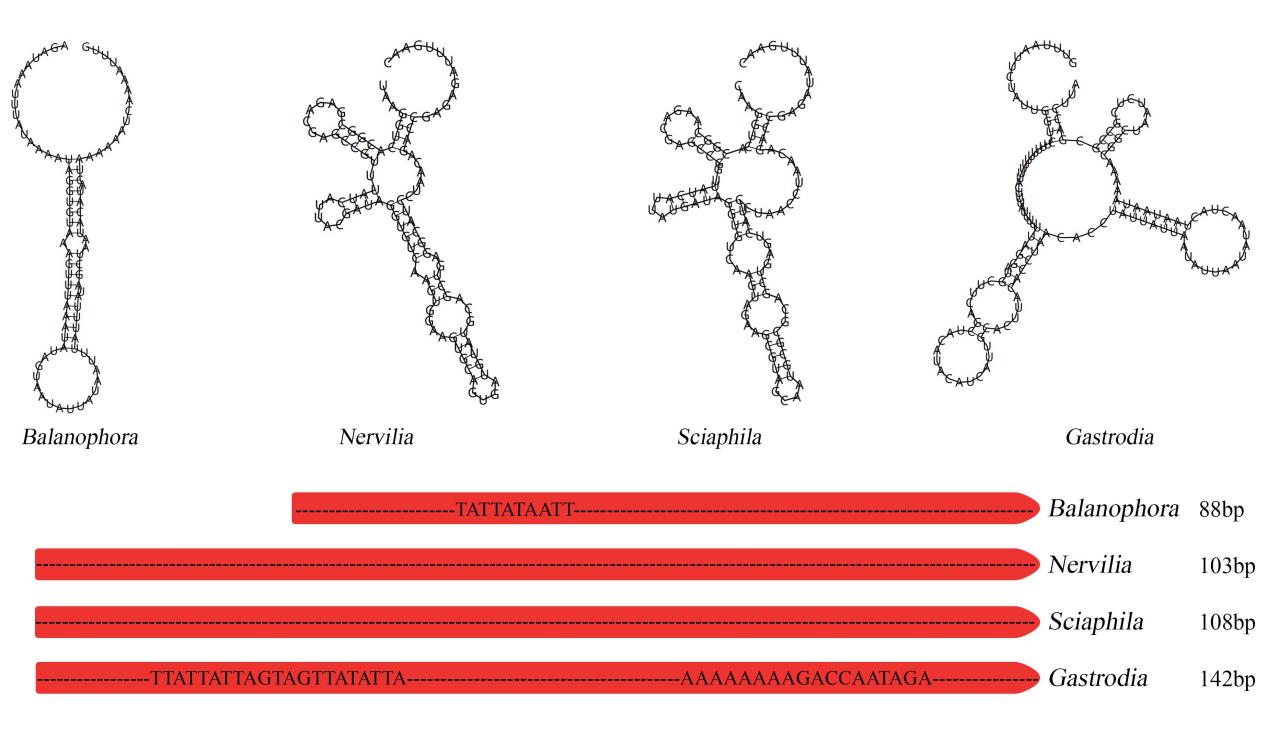


**Supplementary Fig. S3** Secondary structure of the 4.5S rRNA in four species. There are AT-rich inserts in *rrn4.5* of *Balanophora* and *Gastrodia*.


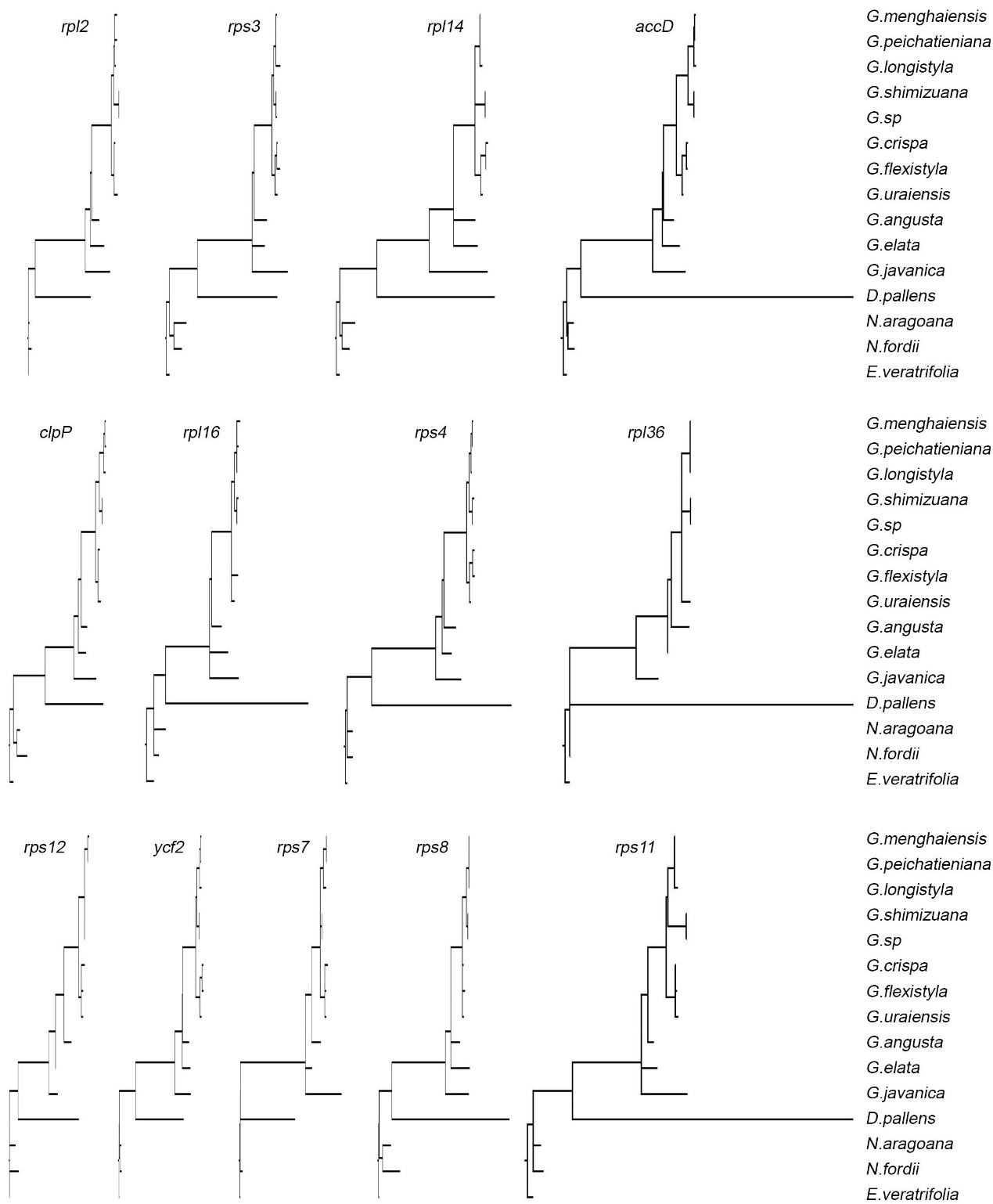


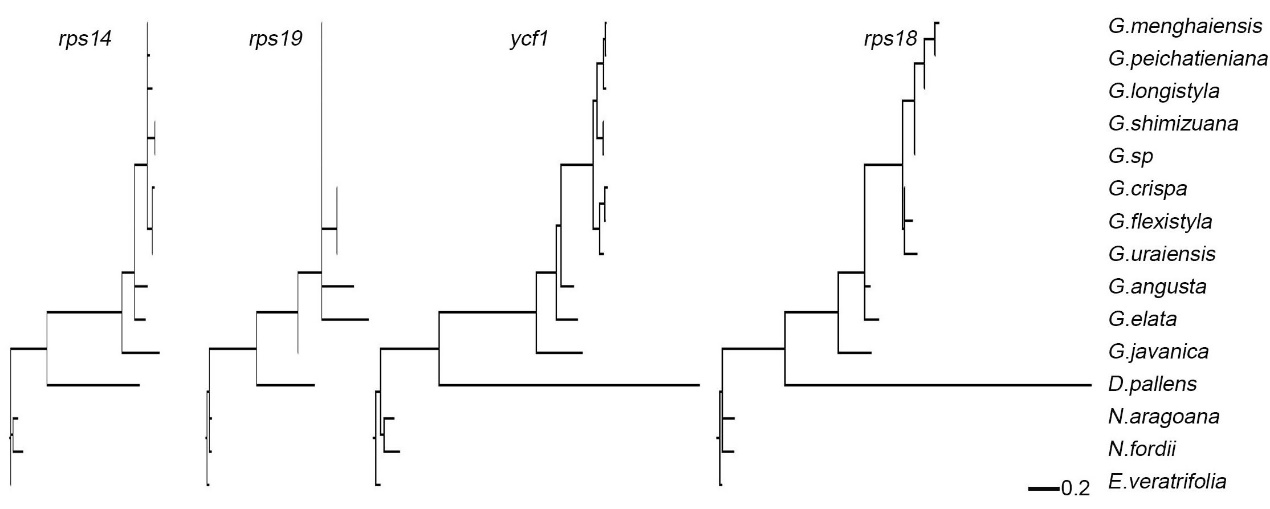


**Supplementary Fig. S4** Synonymous substitution rates of 17 plastid protein-coding genes. All trees are drawn in the same scale.


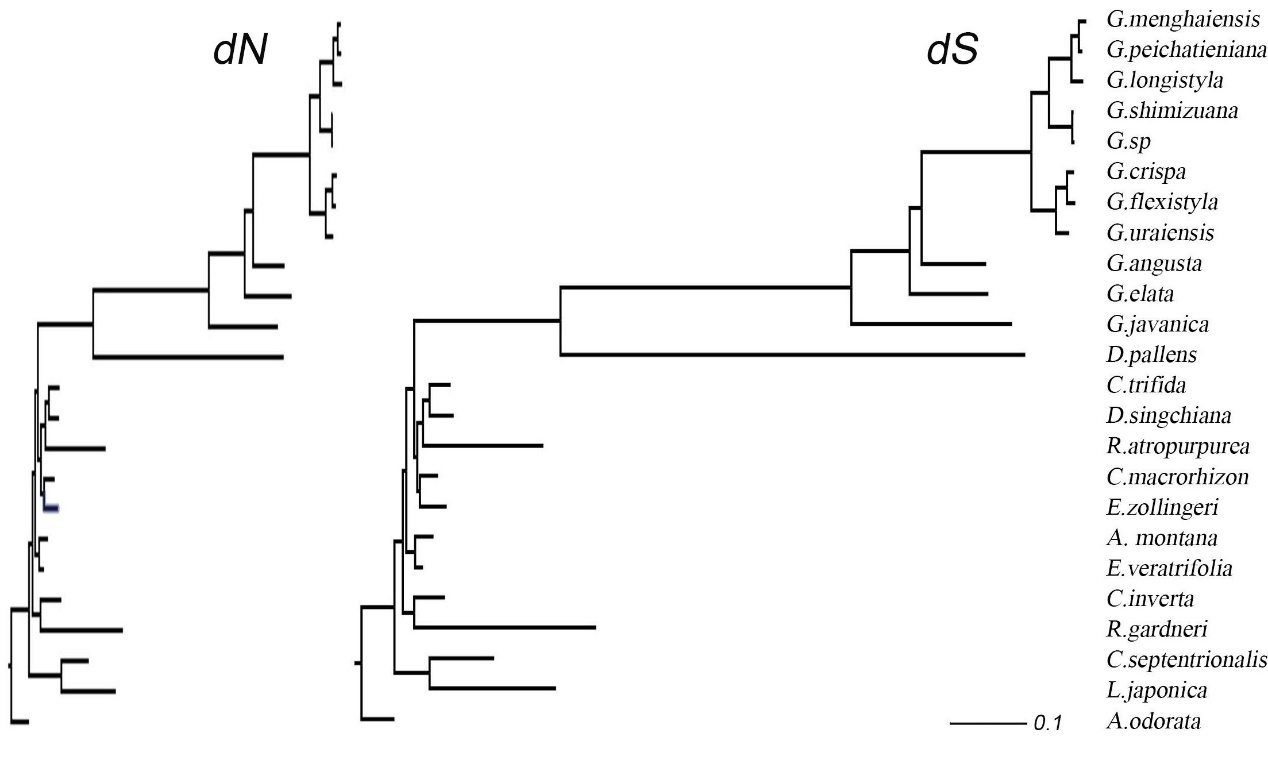


**Supplementary Fig. S5** Synonymous (*dS*) and nonsynonymous (*dN*) substitution rates of plastid genomes with common protein-coding sequences


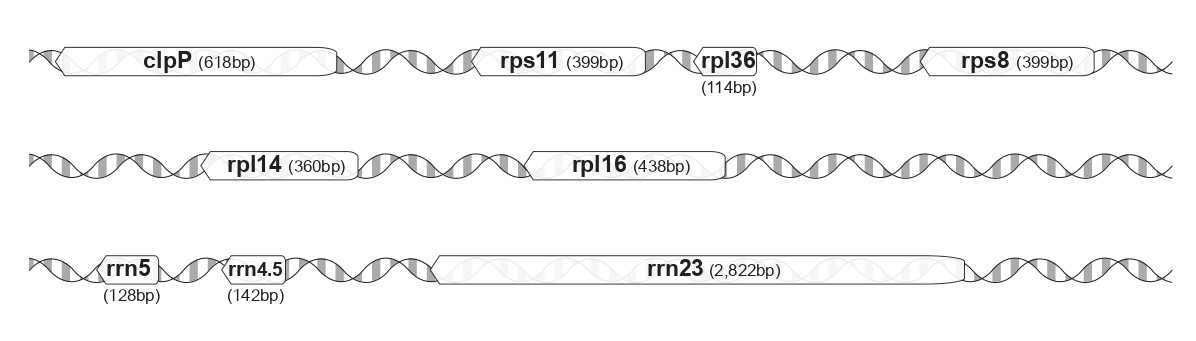


**Supplementary Fig. S6** Transcriptomes of *rrn*, *clpP-rps11-rpl36-rps8*, and *rpl14-rpl16* block of *Gastrodia elata*
